# A prospective “test‐and‐treat” demonstration project among people who inject drugs in Vietnam

**DOI:** 10.1002/jia2.25151

**Published:** 2018-07-09

**Authors:** Hai H Nguyen, Duong D Bui, Thuy TT Dinh, Loc Q Pham, Van TT Nguyen, Tram H Tran, Thang H Pham, Sang M Nguyen, Amitabh B Suthar, Nhan T Do, Nathan Ford, Ying‐Ru Lo, Long Hoang Nguyen, Le M Giang, Masaya Kato, the team for the implementation research project “Vietnam HIV early antiretroviral therapy with regular HIV testing (V‐HEART)”

**Affiliations:** ^1^ Viet Nam Authority of HIV/AIDS Control Ministry of Health Hanoi Vietnam; ^2^ Hanoi Medical University Hanoi Vietnam; ^3^ Vietnam Country Office World Health Organization Hanoi Vietnam; ^4^ National Institute of Hygiene and Epidemiology Hanoi Vietnam; ^5^ HIV Department World Health Organization Geneva Switzerland; ^6^ Regional Office for the Western Pacific World Health Organization Manila Philippines

**Keywords:** antiretroviral therapy, people who inject drugs, Vietnam, viral suppression, retention, test‐and‐treat, risk behaviour

## Abstract

**Introduction:**

Modelling suggests that early diagnosis and immediate antiretroviral therapy (ART) among key populations would have a substantial impact in reducing HIV transmission and mortality in Vietnam. An implementation research project of “test‐and‐treat” among people who inject drugs (PWID) was developed to inform effective roll‐out of such interventions.

**Methods:**

“Test‐and‐treat” was offered to PWID in two high burden provinces, Thai Nguyen and Thanh Hoa. The interventions comprised the offer of biannual HIV testing and immediate ART, irrespective of CD4 count. PWID were enrolled between April 2014 and July 2015 and followed up for 12 months, and retention, HIV viral load (VL) and risk behaviours were assessed. Retention in care of this prospective cohort was compared with the retention among men enrolled in care in the preceding period (April 2012 to March 2013) at the same clinics when ART was initiated at CD4 cell count ≤350 cells/mm^3^.

**Results:**

In total, 287 HIV positive PWID started immediate ART. The majority (98%) were men; median age was 34; and median (interquartile range) CD4 count was 199 (50 to 402) cells/mm^3^. After 12 months, 238 participants (83%) were retained on ART, and 205 achieved viral suppression (<1000 copies/mL) (92% among those in whom VL was measured, 71% overall). Baseline CD4 count ≤100 cells/mm^3^ and history of imprisonment were associated with lower retention and viral suppression, while engagement in methadone maintenance was associated with higher retention. Retention in care was higher in the “test‐and‐treat” cohort (83%) compared with men enrolled in care in the preceding period (78%), primarily because lost‐to‐follow‐up during pre‐ART care was eliminated. No decline in consistent condom use and clean needle use was observed.

**Conclusions:**

Early ART initiation resulted in successful treatment outcomes among PWID, with no observed increase in self‐reported risk behaviours, suggesting feasibility and potential effectiveness of “test‐and‐treat” approach. The results also call for differentiated care for PWID, including promoting early diagnosis and engagement in methadone maintenance therapy while enhancing care for those with advanced HIV disease and history of imprisonment.

## 1 Introduction

Vietnam's HIV epidemic has been concentrated in key populations, with injection drug use being the dominant mode of transmission, like in many Asian and Eastern European countries 1. While HIV prevalence in people who inject drugs (PWID) has declined substantially, national sentinel surveillance in 2016 reported HIV prevalence in PWID was still the highest (11.0%), among the populations surveyed 1. Modelling suggests that, in 2016, 44% of estimated new infections occurred among male PWID (Ministry of Health, unpublished data). There were an estimated 250,000 people living with HIV (PLHIV) 1 and estimated 270,000 PWID 1 in 2016. In the same year, 115,927 people were receiving antiretroviral therapy (ART), representing an estimated 47% of all PLHIV 1, 2.

Results of the HPTN052 trial in 2011 3 showing that early initiation of ART could efficaciously prevent linked HIV transmission in serodiscordant couples inspired discussions in Vietnam about how best to harness such ART benefits in resource‐limited settings. Three modelling studies using data from Vietnam consistently reported that early initiation of ART would avert a substantial number of new HIV infections and AIDS deaths, and that prioritizing key populations for early HIV diagnosis and immediate access to ART is a cost‐efficient strategy for controlling the HIV epidemic 4, 5, 6. One of these studies projected that prioritizing PWID for testing and immediate ART reduces AIDS deaths by 42% and new infections by 70% compared to standard ART scale‐up, suggesting that the highest HIV transmission occurs among PWID, and from PWID to their sexual partners 4.

However, the programmatic experiences of “test‐and‐treat” had been limited, especially in key populations in resource‐limited settings 7. In 2013 when the project planning began, national guidelines recommended ART initiation at CD4 count ≤350 cells/mm^3^ or with clinical stage 3 or 4 diseases. Concerns were raised among national stakeholders to offer ART irrespective of CD4 count among asymptomatic PWID, with regard to adherence, retention and potential increase in risk behaviours. In reality, a number of studies have shown that access to ART for HIV‐positive PWID remains a challenge globally 8, 9, 10. In Vietnam, data have shown that PWID access ART later than HIV‐positive individuals without a history of injecting drug use 11, 12, 13.

In such context, this implementation research project was designed with aims to assess feasibility and potential effectiveness of providing “test‐and‐treat” among PWID and to inform optimization of related policy development and programme implementation.

## 2 Methods

### 2.1 Study design, sites and participants

The study used a prospective cohort design, with viral suppression, retention and self‐reported risk behaviours as primary outcomes of interest, and sample size needed for prospective cohort was initially estimated as 306. Historical comparison of care retention before and after the introduction of “test‐and‐treat” was conducted, where data prior to “test‐and‐treat” were obtained retrospectively.

The study was conducted in Thai Nguyen and Thanh Hoa provinces in Vietnam. HIV prevalence in PWID has been high in both provinces, with some decline in Thai Nguyen (from 38.8% in 2012 to 23.0% in 2016) and fluctuation in Thanh Hoa (11.0% in 2012 to 13.0% in 2016), according to the national HIV sentinel surveillance. HIV testing twice a year was recommended among PWID in both provinces. Immediate ART was offered at 13 HIV outpatient clinics (six in Thai Nguyen, and seven in Thanh Hoa, Table S2). Methadone maintenance therapy (MMT) was available at most districts where study clinics were located (Table S2).

Eligible participants were PWID meeting one of the following conditions: (1) self‐reporting ever injecting any illicit drug and having a visible injection site on the body, (2) self‐reporting injecting illicit drugs in the past month, or (3) receiving MMT. Other eligibility criteria included: ART naïve; aged ≥18 years; living in one of the two study provinces; and no plan to move outside the province for at least one year. Exclusion criteria were: unable to provide consent; acute clinical condition (e.g. cryptococcal meningitis); or participation in another study with a similar research focus. Participants were recruited from April 2014 to July 2015 among those newly diagnosed HIV positive and linked to study clinics, and those who had previously diagnosed and were receiving care but had not been on ART at the clinics.

### 2.2 Interventions

#### 2.2.1 HIV testing services

HIV testing services (HTS) was offered at healthcare facilities and MMT services and through community outreach at selected hot‐spots and remote communes. To promote early HIV diagnosis, healthcare workers and peer educators were trained to promote knowledge of ART and potential benefits and risks for immediate ART among PWID. PWID were recommended to seek HTS once every six months. PWID diagnosed HIV positive were referred to the HIV clinics by healthcare workers or peer educators.

#### 2.2.2 Study enrolment

People were assessed for the eligibility at the study clinics, and if eligible, they received baseline assessment and counselling upon written informed consent. The importance of using prevention measures was emphasized, including condoms, clean needles and MMT as appropriate. Participants then received CD4 cell count and viral load (VL) tests.

#### 2.2.3 ART initiation and follow‐up

Upon study enrolment, participants were provided ART irrespective of CD4 count. Tenofovir, lamivudine and efavirez, as a fixed‐dose combination, was the preferred first‐line regimen. The study followed participants for 12 months from the date ART was started, or until they were recorded as attrition (i.e. death, lost‐to‐follow‐up (LTFU)), whichever occurred earlier. In instances when a participant reported moving to another province (transfer‐out), was arrested or was taken to compulsory drug treatment centres (06 centres) 14, follow‐up was terminated and the individual was excluded from the analysis. In such cases, clinic staff made best efforts to ensure continuity of treatment. In addition, individuals received standard care following the national guidelines, including opportunistic infection prophylaxis and treatment, tuberculosis screening, CD4 count test (every six months), adherence support and ARV drug substitution and switch when needed.

### 2.3 Data collection

Data on key dates (e.g. ART start, attrition, transfer‐out) and treatment outcomes (e.g. VL, CD4 cell count) are recorded on study specific logbook. VL was measured at six and 12 months after ART initiation. The quantitation of HIV‐1 viral ribonucleotide (RNA) were performed using COBAS^®^ AmpliPrep^®^/COBAS^®^ TaqMan^®^ 48 analyser (Roche Molecular Systems Inc., Branchburg, NJ, USA) at National Institute of Hygiene and Epidemiology. Behaviour assessment was offered through counselling sessions, using the standard questionnaire at three, six and 12 months.

To compare retention in care (including pre‐ART and ART care) before and after the introduction of “test‐and‐treat,” data of pre‐“test‐and‐treat” cohort were retrospectively extracted from medical charts at the study clinics. The data on attrition (deaths, LTFU) during the first 12 months after care enrolment, together with injection drug use status if documented on the chart, were obtained for patients who were newly enrolled in care at the study clinics from April 2012 to March 2013. This timeframe was selected to ensure 12 month follow‐up period before “test‐and‐treat” was introduced in April 2014. Individuals who were transferred out or arrested were excluded from the analysis to ensure the consistent inclusion criteria with the prospective cohort.

### 2.4 Data analysis

Data were entered into Epi Info™ 7.0 (Center for Disease Control and Prevention, Atlanta, GA, USA) and analysed using STATA 12.1 (StataCorp LLC, College Station, TX, USA). The proportion of viral suppression, defined as VL <1000 copies/mL following the national and WHO guidelines 15, was analysed both among all those who started ART (intention‐to‐treat) and among those who had VL measured at 12 months. Participants were considered being engaged in MMT when they reported at least twice in their behavioural assessments offered at baseline, month 3, month 6 or month 12.

Cox proportional hazard models, using scaled Schoenfeld residuals to test the proportional hazard assumptions, was applied to analyse correlates for treatment retention, and Poisson regression analysis, with robust error variances 16, was used to analyse correlates for lack of viral suppression at 12 months. Covariates were selected *a priori* based on prior knowledge of potential correlates. Chi square test or Fisher's exact test were used to compare the status at 12 months after the care enrolment between the pre‐ and post‐“test‐and‐treat” cohorts.

In the historical comparison, to compare with the prospective cohort, two comparators were defined for the pre‐“test‐and‐treat” cohort: all the male patients, and those with injection drug use status documented in their medical chart. This was because under‐reporting of the injection drug use status has been common in Vietnam, due to stigma, discrimination and fear of being arrested 11, 12, 13, and PWID represent the majority (60% to 70%) of men attending HIV care 5, 14.

### 2.5 Ethics

The study protocols were reviewed and approved by the Institutional Review Board of Hanoi University of Public Health, and the Ethical Review Committee of the World Health Organization Regional Office for the Western Pacific.

## 3 Results

### 3.1 Study participants and their baseline characteristics

**Figure 1:**
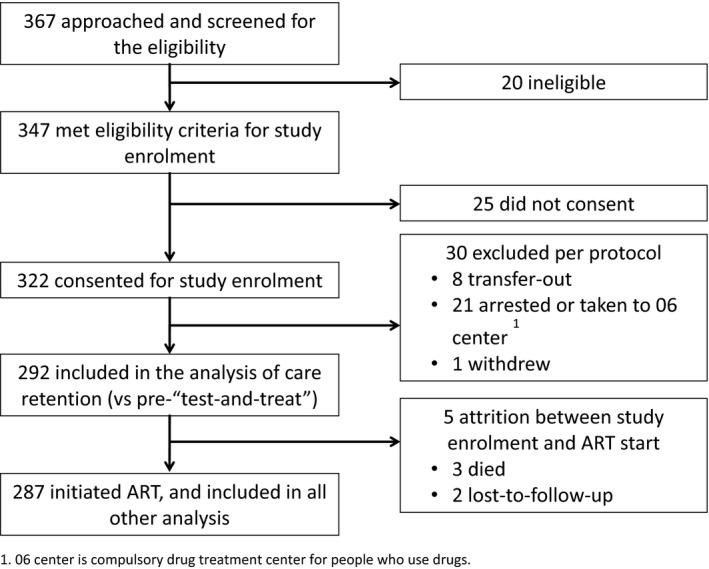
Participant recruitment and inclusion in the analysis. 06 Centre is compulsory drug treatment centre for people who use drugs.

Three hundred sixty‐seven persons were assessed for potential enrolment in the study, among whom 322 individuals met the eligibility criteria, and consented for enrolment; 30 were excluded as per protocol due to transfer‐out, arrest, or withdrawal from the study, resulting in 292 persons included in the comparison of care retention before and after introduction of “test‐and‐treat.” An additional five cases were excluded, due to attrition before ART initiation, resulting in 287 individuals for the analysis of treatment retention, viral suppression and risk behaviours of the prospective cohort (Figure 1).

Table 1 shows the sociodemographic, behavioural and clinical characteristics at enrolment of the 322 individuals who consented to study enrolment, and the 287 individuals who started ART. There were no significant differences in any of measured parameters between the two groups. Of the 287, 98% were males; median (interquartile range, IQR) age was 34 (30 to 39); 58% had monthly income <2 million Vietnamese Dong (approximately US$90); 58% reported injecting opium or heroin in the past month; and 32% reported receiving MMT. At enrolment, 285 and 282 had their CD4 count and VL measured, respectively. Median (IQR) CD4 count was 199 (50 to 402) cells/mm^3^ with 34% having a CD4 count <100 cells/mm^3^. Median (IQR) VL was 61,200 (15,400 to 154,000) copies/mL. Median (IQR) time from study enrolment to ART initiation was 2 (0 to 14) days. The characteristics at care enrolment of PWID in “test‐and‐treat” cohort and men and self‐reporting PWID enrolled in care before “test‐and‐treat” were shown in the Table S3.

**Table 1:** Sociodemographic, behavioural and clinical characteristics at study enrolment of the participants who consented for enrolment and who were included in the analysis

	Eligible and consented for enrolment N = 322 (%)	Those initiated immediate ART N = 287 (%)
Sex		
Female	5 (1.6)	5 (1.7)
Male	317 (98.4)	282 (98.3)
Age		
18 to 29	66 (20.5)	51 (17.8)
30 to 39	185 (57.5)	171 (59.6)
≥40	71 (22.0)	65 (22.6)
Province		
Thai Nguyen	153 (47.5)	137 (47.7)
Thanh Hoa	169 (52.5)	150 (52.3)
Distance from house to clinic (km)		
0 to 9	127 (39.4)	109 (38.0)
10 to 49	156 (48.5)	143 (49.8)
≥50	39 (12.1)	35 (12.2)
Settings of attending HIV clinic^a^		
Urban	160 (49.7)	144 (50.2)
Rural	133 (41.3)	121 (42.1)
Remote	29 (9.0)	22 (7.7)
Monthly income (Vietnam Dong)		
No income	56 (17.4)	46 (16.0)
<2 million	135 (41.9)	119 (41.5)
≥2 million	131 (40.7)	122 (42.5)
Employment		
Unemployed	61 (18.9)	52 (18.1)
Employed	261 (81.1)	235 (81.9)
Occupation		
Farmer	122 (37.9)	107 (37.3)
Self‐employed	122 (37.9)	112 (39.0)
Others	26 (8.1)	25 (8.7)
Education		
Illiterate	8 (2.5)	7 (2.5)
Primary school	53 (16.6)	46 (16.1)
Secondary school	148 (46.4)	133 (46.7)
High school	98 (30.7)	87 (30.5)
University/College	12 (3.8)	12 (4.2)
Marital status		
Unmarried	112 (34.8)	90 (31.4)
Married	210 (65.2)	197 (68.6)
Ever in prison		
Yes	91 (28.3)	80 (27.9)
No	231 (71.7)	207 (72.1)
Ever in compulsory drug treatment centre		
Yes	76 (23.6)	59 (20.6)
No	246 (76.4)	228 (79.4)
Consistent condom use with regular partner in the past three months (baseline)		
Yes	90 (66.7)	83 (66.4)
No	45 (33.3)	42 (33.6)
Service use in the past three months (baseline)		
Needle syringe programme	43 (13.4)	37 (12.9)
Methadone maintenance therapy	105 (32.6)	92 (32.1)
CD4 count before ART start (count/mm^3^)		
N	319	285
Median (IQR)	202 (58 to 402)	199 (50 to 402)
Viral load before ART start (copies/mL)		
N	316	282
Median (IQR)	59650 (13,750 to 150,500)	61200 (15,400 to 154,000)

ART, antiretroviral therapy; IQR, interquartile range.

^a^Clinics in the provincial capitals were categorized as urban, those in two mountainous districts are categorized as remote, and the rest were categorized as rural.

### 3.2 Retention and viral suppression of prospective cohort

**Figure 2:**
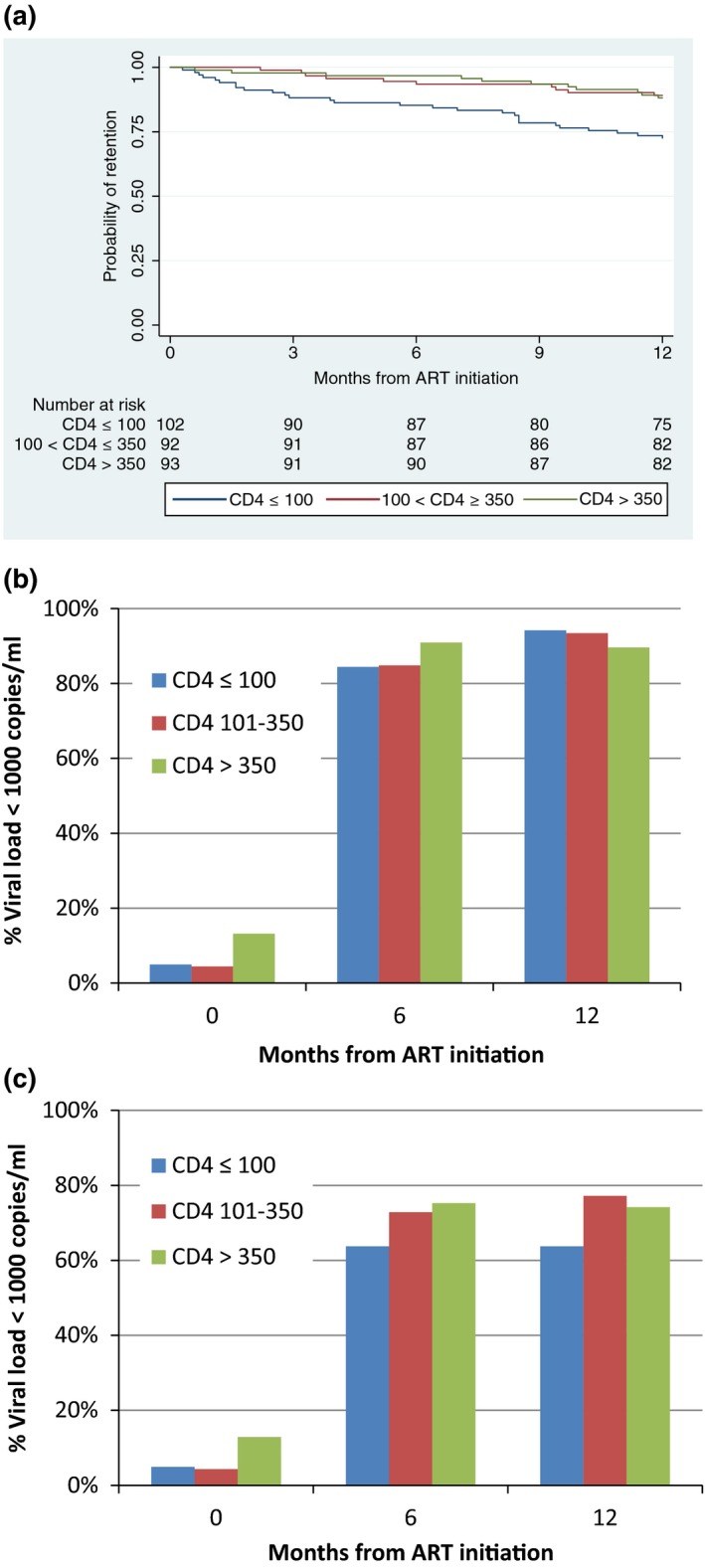
Retention on treatment and proportion achieving viral suppression among HIV‐positive people who inject drugs disaggregated by CD4 count. **(a)** Kaplan‐Meier curve showing the proportion of the individuals retained on treatment by baseline CD4 count. Retention at 12 months were 88.2%, 89.1% and 72.6% among those starting antiretroviral therapy (ART) with CD4 count >350 cells/mm^3^ (N = 93), between 101 and 350 cells/mm^3^ (N = 92) and ≤100 cells/mm^3^ (N = 102). **(b,c)** The proportion of the individuals achieving viral suppression (<1000 copies/mL) at month 0, 6 and 12 of ART among those tested for viral load (b, N = 282, 233 and 222 at month 0, 6 and 12 respectively) and among all those who started ART (c, N = 287).

Of the 287 individuals starting ART, 83% were still on treatment 12 months after ART initiation (Figure 2a). Those with baseline CD4 count >350 cells/mm^3^ (N = 93) and between 101 and 350 cells/mm^3^ (N = 92) had significantly higher retention (88.2%, *p *= 0.007 and 89.1%, *p *= 0.005, respectively) than those with baseline CD4 count ≤ 100 cells/mm^3^ (N = 102, 72.5%). Forty‐nine persons had attrition (28 deaths and 21 LTFU), with the majority of deaths (20 cases) occurring in the first six months, while majority of LTFU (17 cases) being recorded in the second six months.

Of those who had VL measured before and at six and 12 months after ART start (N = 282, 233 and 222 respectively), the proportion VL suppressed (<1000 copies/mL) was 8%, 87% and 92% respectively. There was no significant difference in the proportion achieving viral suppression at 12 months among the groups with different baseline CD4 count levels (≤100, 101 to 350, >350) (*p* = 0.53) (Figure 2b). If all those who started ART were included in the denominator at each time point (intention‐to‐treat, N = 287), the proportion of the individuals with VL <1000 copies/mL was 7%, 70% and 71% at the month 0, 6 and 12, respectively. Those with baseline CD4 count >350 and at 101 to 350 cells/mm^3^ had higher proportion achieving viral suppression at 12 month (74.2%, *p* = 0.077 and 77.2%, *p* = 0.014 respectively) than those with ≤100 cells/mm^3^ (63.7%) (Figure 2c). The clinics failed to obtain specimens on time for VL measurement for 5 (1.7%) at baseline, and 30 and 16 at months 6 and 12 (10.2% and 6.3% among retained) respectively.

### 3.3 Correlates of treatment retention and viral suppression

In the multivariate analysis, baseline CD4 count from 101 to 350 cells/mm^3^ (adjusted hazard ratio, aHR 0.41, 95% CI 0.19 to 0.87) and >350 cells/mm^3^ (aHR 0.35, 95% CI 0.17 to 0.74) compared with CD4 ≤ 100 cells/mm^3^ and engagement in MMT (aHR 0.34, 95% CI 0.16 to 0.75) were associated with lower attrition, and history of imprisonment was associated with greater attrition (aHR 2.94, 95% CI 1.46 to 5.94) (Table 2). For viral suppression, baseline CD4 count from 101 to 350 cells/mm^3^ (adjusted risk ratio, aRR 0.53, 95% CI 0.31 to 0.90) and >350 cells/mm^3^ (aRR 0.57, 95% CI 0.35 to 0.91) compared with CD4 < 100 cells/mm^3^ were associated with lower risk of lack of viral suppression (based on intention‐to‐treat analysis), and history of imprisonment were associated with lack of viral suppression (aRR 1.90, 95% CI 1.21 to 2.99) (Table 2).

**Table 2:** Factors associated with attrition in the first 12 months after ART and lack of viral suppression at 12 months after ART initiation

	Hazard ratio for attrition from ART during the 12 month				Risk ratio for non‐viral suppression at 12 month after ART start (intention‐to‐treat)			
	HR (95% CI)	*p*	aHR (95% CI)	*p*	RR (95% CI)	*p*	aRR (95% CI)	*p*
Sex								
Female	1		1		1		1	
Male	0.80 (0.11 to 5.80)	0.826	1.32 (0.17 to 10.10)	0.791	1.22 (0.21 to 7.17)	0.824	0.64 (0.19 to 2.19)	0.482
Age								
18 to 29	1		1		1		1	
30 to 39	1.08 (0.49 to 2.38)	0.841	1.20 (0.53 to 2.73)	0.663	0.82 (0.45 to 1.52)	0.534	1.28 (0.71 to 2.30)	0.410
≥40	1.35 (0.56 to 3.25)	0.508	1.45 (0.56 to 3.77)	0.441	0.97 (0.58 to 1.62)	0.913	1.18 (0.59 to 2.37)	0.635
Distance from house to clinic								
0 to 9	1				1			
10 to 49	0.96 (0.53 to 1.76)	0.905			0.61 (0.39 to 0.96)	0.034		
≥50	0.96 (0.38 to 2.39)	0.924			0.75 (0.38 to 1.47)	0.401		
Setting of clinic								
Urban	1		1		1		1	
Rural	0.51 (0.28 to 0.94)	0.031	0.72 (0.37 to 1.40)	0.329	0.70 (0.45 to 1.09)	0.111	0.88 (0.56 to 1.40)	0.587
Remote	0.18 (0.03 to 1.34)	0.094	0.29 (0.04 to 2.19)	0.231	0.16 (0.02 to 1.10)	0.062	0.21 (0.03 to 1.41)	0.109
Income (Vietnamese Dong)								
No income	1		1		1		1	
<2 million	0.45 (0.22 to 0.93)	0.030	1.40 (0.47 to 4.17)	0.550	0.66 (0.37 to 1.17)	0.158	1.41 (0.63 to 3.16)	0.402
≥2 million	0.50 (0.25 to 1.01)	0.054	1.40 (0.46 to 4.23)	0.551	0.86 (0.50 to 1.47)	0.578	1.74 (0.73 to 4.15)	0.213
Employment status								
Unemployed	1		1		1		1	
Employed	0.41 (0.23 to 0.75)	0.003	0.58 (0.21 to 1.64)	0.306	0.65 (0.41 to 1.03)	0.068	0.68 (0.32 to 1.45)	0.316
Education status								
High school or less	1		1		1		1	
High school or College	0.63 (0.33 to 1.19)	0.156	0.69 (0.35 to 1.33)	0.266	0.86 (0.55 to 1.36)	0.530	0.82 (0.51 to 1.31)	0.410
Marital status								
Unmarried	1		1		1		1	
Married	0.52 (0.30 to 0.92)	0.023	0.69 (0.36 to 1.32)	0.268	0.62 (0.41 to 0.94)	0.024	0.73 (0.47 to 1.14)	0.167
CD4 count (baseline)								
≤100	1		1		1		1	
101 to 350	0.35 (0.17 to 0.73)	0.005	0.41 (0.19 to 0.87)	0.021	0.53 (0.31 to 0.91)	0.021	0.53 (0.31 to 0.90)	0.019
>350	0.38 (0.19 to 0.77)	0.007	0.35 (0.17 to 0.74)	0.006	0.65 (0.40 to 1.07)	0.090	0.57 (0.35 to 0.91)	0.020
Level of consuming alcohol in the past month (baseline)								
None	1				1			
Occasionally	0.66 (0.36 to 1.22)	0.187			0.81 (0.52 to 1.28)	0.370		
Daily	0.76 (0.33 to 1.71)	0.501			0.73 (0.37 to 1.42)	0.349		
Engagement in MMT								
No	1		1		1		1	
Yes	0.38 (0.18 to 0.77)	0.008	0.34 (0.16 to 0.75)	0.007	0.95 (0.62 to 1.46)	0.821	0.93 (0.57 to 1.54)	0.789
Ever in prison								
No	1		1		1		1	
Yes	2.51 (1.43 to 4.01)	0.001	2.94 (1.46 to 5.94)	0.003	1.96 (1.30 to 2.95)	0.001	1.90 (1.21 to 2.99)	0.006
Ever in compulsory drug treatment centre								
No	1				1			
Yes	0.61 (0.27 to 1.36)	0.222			0.76 (0.43 to 1.36)	0.356		

Hazard ratio for attrition from ART during the first 12 month was estimated through cox proportional hazard model. Risk ratio for non‐suppression of VL at 12 month after ART start was obtained through Poisson regression, including all those who started ART in the analysis, except those who were retained but missed VL test at month 12 (intention‐to‐treat). Two million Vietnamese Dong was approximately US$90.

MMT, methadone maintenance therapy; ART, antiretroviral therapy; VL, viral load.

### 3.4 Self‐reported risk behavior

Among respondents retained and reporting ongoing injection of opium or heroin (N = 166, 128, 105 and 79 at month 0, 3, 6 and 12 respectively), a high proportion reported using clean needles at their last injection (90%, 89%, 95% and 92% respectively). Of respondents reporting sexual intercourse with their regular partner in the past three months (N = 125, 120, 118 and 110 at month 0, 3, 6 and 12 respectively), the proportion reporting consistent condom use in the past three months showed an increasing trend (66%, 84%, 90% and 90% respectively).

### 3.5 Historical comparison of care retention before and after introduction of “test‐and‐treat”

**Figure 3:**
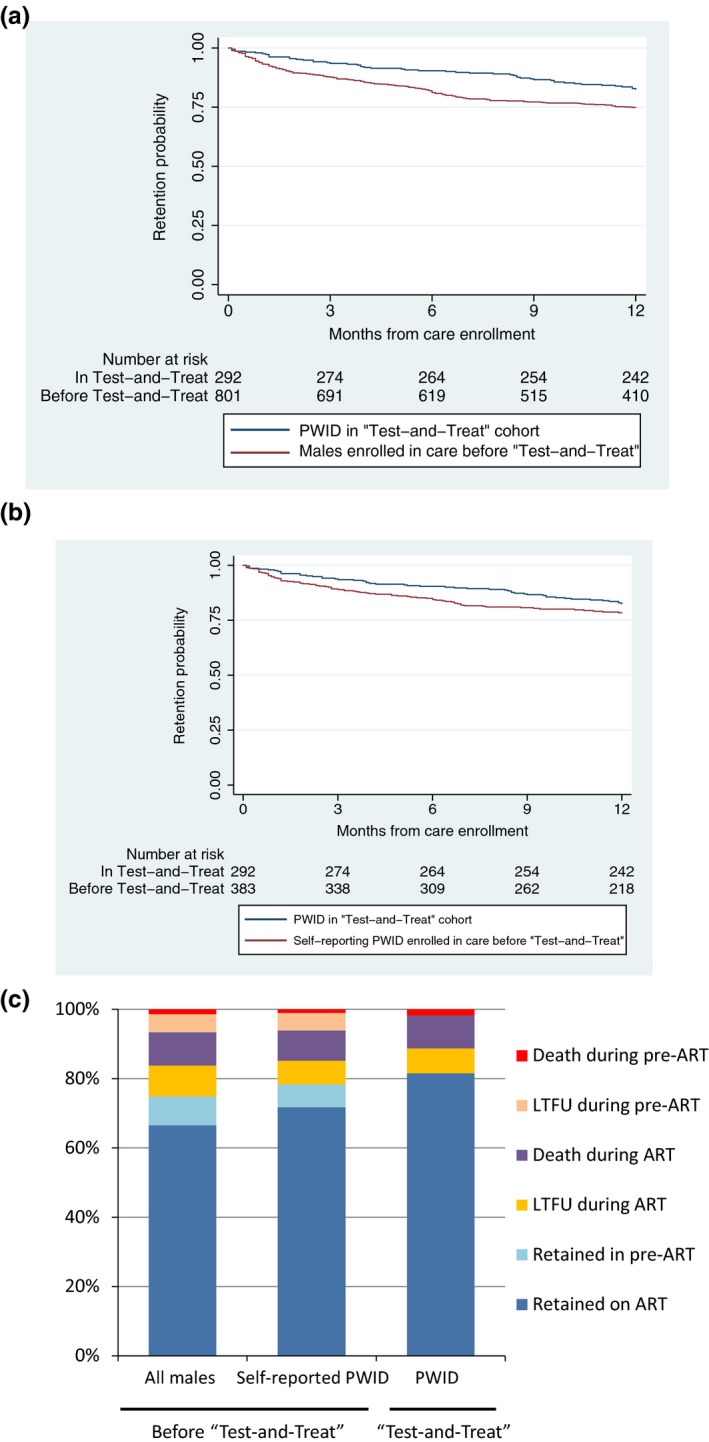
Comparison of retention in care before and after introduction of “test‐and‐treat.” **(a)** Retention among all the males enrolled in care prior to “test‐and‐treat” and people who inject drugs (PWID) enrolled in the “test‐and‐treat” cohort. **(b)** Retention among self‐reporting PWID enrolled in care prior to “test‐and‐treat” and PWID enrolled in the “test‐and‐treat” cohort. **(c)** 12 month outcomes of all the males and self‐reporting PWID enrolled in care prior to “test‐and‐treat” and PWID enrolled in the “test‐and‐treat” cohort. Data on men (a, N = 801) and self‐reported PWID (b, N = 375) enrolled in care prior to introduction of “test‐and‐treat” were retrospectively extracted from medical charts at the same clinics. Retention in care was significantly higher in PWID enrolled in the “test‐and‐treat” cohort (N = 292) than in men enrolled in care prior to “test‐and‐treat” (82.5% vs. 74.9% at 12 months after care enrolment), but the difference with self‐reported PWID (78.3% at 12 months) was insignificant.

Retention was significantly higher among PWID in “test‐and‐treat” cohort (N = 292), than among the males enrolled in care prior to introduction of “test‐and‐treat” (N = 801, *p* = 0.004) (Figure 3a). When the prospective cohort was compared with those whose PWID status was documented on medical records (N = 383), retention tended to be higher in the former than the latter, although the difference was not statistically significant (*p* = 0.128). Figure 3c shows status at 12 months after care enrolment, comparing the PWID in the prospective “test‐and‐treat” cohort, and all males and self‐reporting PWID enrolled in care prior to “test‐and‐treat.” There were no significant differences in the number of deaths during pre‐ART and ART care, and LTFU during ART; however, LTFU during pre‐ART care was significantly lower (*n* = 2, 0.7%) in the former than the latter (*n* = 39, 4.9%) (*p* < 0.001). The result was consistent when those with PWID status documented was used as the comparator (*n* = 18, 4.8% LTFU during pre‐ART, *p* = 0.002).

## 4 Discussion

In this demonstration project, conducted in two Vietnamese provinces affected by heroin injection and associated HIV transmission, PWID were able to achieve relatively high retention (83%) and viral suppression (92% among tested) in the first 12 months of ART without increasing self‐reported risk behaviors. The “test‐and‐treat” approach also significantly reduced LTFU associated with pre‐ART care. These results suggest that “test‐and‐treat” among PWID will provide programmatically feasible and potentially high impact interventions, and likely contribute in effectively controlling HIV epidemic. Based on the interim results of this study, combined with other emerging evidence, the Ministry of Health of Vietnam decided to recommend provision of ART irrespective CD4 count for key populations from July 2015.

Our study provided some of the first data on viral suppression among PWID in Vietnam. The level of viral suppression in the present cohort was comparable to outcomes reported by the other previous studies conducted in Vietnam among people on ART in general, including cohort studies 17, 18, a cross‐sectional survey 19 and nationally representative sample of adults receiving ART for 36 months or longer 20. Those studies consistently showed that >90% of PLHIV on ART are achieving viral suppression (<1000 copies) at 12 months or longer on ART. Globally, there have been limited data available on viral suppression among PWID; however, a review study reported that viral suppression among PWID on ART ranged from 42.4% in Thailand to 84.7% in France between 2010 and 2016 21. While careful interpretation is needed due to differences in methods, time period, and duration of follow‐up, PWID in our prospective cohort apparently showed relatively high level of viral suppression compared to those reported from other countries to date.

Our data confirmed that many PWID start ART at an advanced stage of HIV infection (34% had CD4 count ≤100 cells/mm^3^). Attrition (deaths and LTFU) and lack of viral suppression at 12 months were associated with low baseline CD4 count (<100 cells/mm^3^). Late presentation for care and late initiation of ART have been common in Vietnam 12, 13, 22, 23, and PWID have been initiating even with lower CD4 count than non‐PWID populations 11, 12, 13. To harness the full benefits of ART, further efforts are critical to facilitate earlier diagnosis, especially through addressing psychosocial and economic barriers for key populations to access HIV services. Key populations in Vietnam continue to experience stigma and discrimination at healthcare settings and communities 23, 24 and nearly 30% of PLHIV believed that their serostatus was disclosed to others without their consent 24. It is essential to foster more supportive environment and confidential settings for PWID and other key populations to access HIV and other health services.

Attrition from HIV treatment and lack of viral suppression at 12 months were also associated with history of imprisonment. It has been reported that “disproportionately high numbers of the poorest and most excluded are arrested, detained and imprisoned” 25, and thus it is possible that history of imprisonment was associated with unmeasured socioeconomic obstacles that negatively affected HIV treatment outcomes. Studies in high‐income settings reported that provision of education and case management services improve retention in HIV care after release from jail 26 and models for improving linkage to care for PLHIV released from jail or prison have been proposed 27. Locally feasible and effective models should be developed to improve HIV treatment outcomes for the PWID with imprisonment history.

Consistent with other findings, engagement in MMT was independently associated with higher ART retention. A recent meta‐analysis based on 32 studies, mostly from high income countries, reported that opioid substitution therapy was associated with an increase in recruitment onto and adherence to ART, a decrease in the odds of attrition, and improved viral suppression 28. However, our study found no association between engagement in MMT and viral suppression. A cross‐sectional survey of 1255 adult patients on ART for at least one year across four provinces in Vietnam also found no association between the enrolment in MMT and the level of viral suppression 19. It is possible that PWID receiving and not receiving MMT may have different characteristics, and that the effect of MMT on adherence may be mitigated over time 19. Another study in China found that those engaged in MMT had substantially lower mortality rate than those who actively use drugs, while virological failure rate were similar between the two groups 29. In our study, anecdotal information suggested that there is an increasing use of amphetamine‐like stimulants among people who inject heroin and MMT users, which might have had impact on the adherence and viral suppression. Further research is needed to optimize service delivery of MMT and ART among PWID.

These considerations led us to consider that differentiated care approaches should be strengthened for PWID in Vietnam. Differentiated care is an approach that simplifies and adapts HIV services to better serve PLHIV with diverse needs, and reduce unnecessary burdens on the health system 15, 30. First, the WHO recommended package of interventions aimed at reducing HIV‐associated morbidity and mortality should be provided to PWID with advance HIV infection, given high levels of attrition observed among them in our cohort 31. Second, PWID with past history of imprisonment should be identified and provided with package of care and psychosocial support to address avoidable attrition. Third, regular uptake of HIV testing for early diagnosis and access to MMT should be promoted as the core element of services for PWID. While focusing resources for care and support to those populations in need, PWID who are clinically and socially stable may benefit from less frequent clinic visits and follow up at MMT clinics, primary healthcare facilities, or community settings 15, 30. Vietnam is now adopting rapid initiation of ART, including starting ART on the same day as HIV diagnosis for people who are ready, in line with the WHO recommendation 31. This approach is expected to further improve treatment outcomes among PWID.

Our study used combination of self‐report of drug use, identification of visible injection site and engagement in MMT to define PWID, and did not use urine test to confirm the presence of opioid. The primary reasons were to simplify the procedures, assuming future scale‐up of the intervention, and to avoid additional barriers for study participation. Disclosing drug injection status already poses disincentives, as injection drug use has been associated with “social evil,” stigma, and discrimination in Vietnam 23, 32. Adding the requirement for urine test was considered to be an unnecessary additional barrier and may introduce selection bias for PWID to participate in this demonstration project. Although urine testing was not conducted, we believe our definition provides relatively high validity of true PWID population, since self‐report of “ever injecting drugs” appears to be proxy to active injection behaviour, as reported in Vietnam 33, and enrolment into MMT requires strict assessment of heroin injection status including urine test.

Data on treatment outcomes among key populations is scarce, both in Vietnam and in general. Our study measured clinically important outcomes, including VL, among PWID receiving ART. However, we note some limitations. First, we assume that there are potential confounders for the historical comparison of pre‐ and post‐“test‐and‐treat” cohorts, but were unable to control for such confounding as quality data were limited for retrospective analysis. In addition, we used all the males and self‐reporting PWID as comparators to the prospective cohort. Thus, results require careful interpretation, but the important fact is that LTFU associated with pre‐ART care was much fewer after the introduction of “test‐and‐treat” regardless the comparator. Second, VL measurement was missed at some time‐point for some individuals; however, the proportion of missing VL data were relatively small (6.3% at month 12) and it unlikely affects the overall conclusions. Third, the outcomes of patients lost to care are unknown, and could include both positive outcomes (e.g. self‐transfers) and negative outcomes (e.g. deaths). Forth, our study recruited only a few females, and was unable to provide data on female PWID. Finally, our study followed the PWID only for the first 12 months of ART, and further study is needed to assess long‐term durability of immediate ART.

## 5 Conclusion

In conclusion, our study demonstrates that “test‐and‐treat” among PWID is a feasible and effective intervention in Vietnam. Early ART initiation resulted in relatively high retention and viral suppression among PWID without increasing self‐reported risk behaviors, while the “test‐and‐treat” approach reduced the risk of patients being lost during pre‐ART care. The interim results of this study informed revision of the national guidelines to recommend immediate ART among key populations in 2015. In order to achieve full therapeutic and preventive benefits of immediate ART in line with the global and Vietnam's goal to reach 90‐90‐90 by 2020 34, further efforts are essential to strengthen supportive environment for PWID and other key populations to ensure their early diagnosis and immediate ART initiation.

## Competing interests

None to declare.

## Authors’ contributions

MK, TTTD, HHN, VTTN, LMG, DDB, SMN and NTD designed the study, with advice from Y‐RL, NF, LHN and inputs from other co‐authors. Intensive monitoring of project implementation was conducted by HHN, TTTD, LQP, VTTN, THT, THP, SMN, ABS, NTD with support from DDB, LHN, LMG and MK. Data were managed and analysed by LQP, SMN, THT, THP and MK with advice from NF. MK drafted manuscript, with extensive feedback from other co‐authors.

## Supporting information


**Table S1.** List of members of the team for the implementation research project “Vietnam HIV early antiretroviral therapy with regular HIV testing (V‐HEART).”
**Table S2.** List of study sites.
**Table S3.** Characteristics at care enrolment of people who inject drugs in “test‐and‐treat” cohort and men enrolled in care before “test‐and‐treat.”
