# Supplementary material for: A prospective “test‐and‐treat” demonstration project among people who inject drugs in Vietnam
Source: J Int AIDS Soc. 2018 Jul 9;21(7):e25151. doi: 10.1002/jia2.25151 (PMC6036396; doi:10.1002/jia2.25151)
Supplement: Supplementary file 1 — Table S1. List of members of the team for the implementation research project “Vietnam HIV early antiretroviral therapy with regular HIV testing (V‐HEART).” Table S2. List of study sites. Table S3. Characteristics at care enrolment of people who inject drugs in “test‐and‐treat” cohort and men enrolled in care before “test‐and‐treat.” [file JIA2-21-e25151-s001.docx]

# Appendix – Supporting information

## Table S1. List of members of the team for the implementation research project “Vietnam HIV early antiretroviral therapy with regular HIV testing (V-HEART)”

**Principal Investigators**

Bui Duc Duong

Masaya Kato

**Viet Nam Authority for HIV/AIDS Control**

Bui Duc Duong

Nguyen Huu Hai

Do Thi Nhan

Nguyen Hoang Long

**Hanoi Medical University**

Le Minh Giang

Dinh Thi Thanh Thuy

Pham Quang Loc

Nguyễn Minh Sang

Pham Thi Thu Huyen

Tran Quoc Tuan

**National Institute of Hygien and Epidemiology**

Pham Hong Thang

Tran Hong Tram

**Thai Nguyen Provincial AIDS Center**

Le Ai Kim Anh

Ho Thi Quynh Trang

**ThanhHoa Provincial AIDS Center**

Nguyen Ba Can

Vu Dinh Nam

**World Health Organization Viet Nam Country Office**

Masaya Kato

Nguyen Thi Thuy Van

Amitabh Suthar

Vu Quoc Dat (consultant)

Kristine Buchmann (intern)

**World Health Organization Regional Office for the Western Pacific**

Ying-Ru Lo

**World Health Organization HIV Department**

Nathan Ford

## Table S2. List of study sites

| **Province** | **Urban / rural settings** | **City / District** | **MMT availability within each district** | **Name of health facility where HIV outpatient clinic is located** | **The number consented for enrolment**  **N = 322** | **The number started immediate ART**  **N = 287** |
| --- | --- | --- | --- | --- | --- | --- |
| Thai Nguyen | Urban | Thai Nguyen City | Yes | Thái Nguyên Preventive Medicine Center | 43 (13.4) | 38 (13.2) |
|  |  | (Provincial capital) |  | Thái Nguyên Hospital A | 16 (5.0) | 16 (5.6) |
|  | Rural | Phu Binh District | No | Phú Bình | 30 (9.3) | 27 (9.4) |
|  |  | Phu Luong District | Yes | Phú Lương | 30 (9.3) | 25 (8.7) |
|  |  | Dai Tu District | Yes | Đại Từ | 19 (5.9) | 17 (5.9) |
|  |  | Dong Hy | Yes | Đồng Hỷ | 15 (4.7) | 14 (4.9) |
| Thanh Hoa | Urban | Thanh Hoa City  (Provincial capital) | Yes | Thanh Hóa Provincial AIDS Center | 61 (18.9) | 55 (19.2) |
|  |  |  |  | Thanh Hóa City Health Center | 40 (12.4) | 35 (12.2) |
|  | Rural | Bim Son District | Yes ^1^ | Bỉm Sơn | 7 (2.3) | 7 (2.4) |
|  |  | Tho Xuan District | Yes ^1^ | Thọ Xuân | 3 (0.9) | 3 (1.0) |
|  |  | Ngoc Lac District | Yes ^1^ | Ngoc Lac | 29 (9.0) | 28 (9.8) |
|  | Remote  (Mountaneous) | Quan Hoa District | Yes | Quan Hóa | 23 (7.1) | 18 (6.3) |
|  |  | Muong Lat District | Yes | Mường Lát | 6 (1.9) | 4 (1.4) |
|  |  |  |  |  |  |  |

1. In Bim Son, Tho Xuan and Ngoc Lac districts, MMT services were established during the follow-up period of this study.

## Table S3. Characteristics at care enrolment of PWID in “Test-and-Treat” cohort and men enrolled in care before “Test-and-Treat”

|  | **PWID in “Test-and-Treat” cohort** |  | **Those enrolled in care before introduction of “Test-and-Treat”** | |
| --- | --- | --- | --- | --- |
|  | **Those included in the analysis of care retention**  **N = 292 (%)** |  | **All men**  **N = 801 (%)** | **Men with injection drug use status recorded**  **N = 383 (%)** |
| Sex |  |  |  |  |
| Female | 5 (1.7) |  | 0 | 8 (2.1) |
| Male | 287 (98.3) |  | 801 (100) | 375 (97.9) |
| Age group |  |  |  |  |
| < 18 | 0 |  | 6 (0.8) | 0 |
| 18-29 | 53 (18.2) |  | 217 (27.1) | 108 (28.2) |
| 30-39 | 173 (59.2) |  | 424 (52.9) | 202 (52.7) |
| ≥40 | 66 (22.6) |  | 154 (19.2) | 73 (19.1) |
| Province |  |  |  |  |
| Thai Nguyen | 140 (47.9) |  | 389 (48.6) | 138 (36.0) |
| Thanh Hoa | 152 (52.1) |  | 412 (51.4) | 245 (64.0) |
| Initiated ART |  |  |  |  |
| No | 5 (1.7) |  | 77 (9.6) | 33 (8.6) |
| Yes | 287 (98.3) |  | 724 (90.4) | 350 (91.4) |
| CD4 count before ART start (count/mm^3^) |  |  |  |  |
| N | 290 |  | 517 | 256 |
| Median (IQR) | 198 (47 – 402) |  | 92 (31 – 255) | 113 (36 – 285) |

“PWID in “Test-and-Treat” cohort” were enrolled in care from April 2014 to July 2015. “Those enrolled in care before introduction of “Test-and-Treat”” were enrolled in care between April 2012 to March 2013.
